# What we talk about when we talk about reliability: Comment on ‘Intra‐ and inter‐session reliability and repeatability of ^1^H magnetic resonance spectroscopy for determining total creatine concentrations in multiple brain regions’ by Pratt et al.

**DOI:** 10.1113/EP092746

**Published:** 2025-05-05

**Authors:** Rie Skovly Thomsen, Milan Mohammad, Ronan M. G. Berg, Jacob Peter Hartmann, Markus Harboe Olsen

**Affiliations:** ^1^ Centre for Physical Activity Research Copenhagen University Hospital – Rigshospitalet Copenhagen Denmark; ^2^ Department of Clinical Physiology and Nuclear Medicine Copenhagen University Hospital – Rigshospitalet Copenhagen Denmark; ^3^ Department of Cellular and Molecular Medicine, Faculty of Health and Medical Sciences University of Copenhagen Copenhagen Denmark; ^4^ Department of Clinical Medicine, Faculty of Health and Medical Sciences University of Copenhagen Copenhagen Denmark; ^5^ Neurovascular Research Laboratory, Faculty of Life Sciences and Education University of South Wales Pontypridd UK; ^6^ Department of Neuroanaesthesiology, The Neuroscience Centre Copenhagen University Hospital – Rigshospitalet Copenhagen Denmark

**Keywords:** ^1^H magnetic resonance spectroscopy, physiolometrics, reliability

1

We read with great interest the article by Pratt et al., recently published in *Experimental Physiology*, in which proton magnetic resonance spectroscopy (^1^H MRS) was used to quantify total creatine concentrations across several human brain regions (Pratt et al., 2025). The implications of this study are extensive, because it is highly relevant for understanding the fundamental role of brain bioenergetics in humans and how aberrations in this process might contribute to various physiological states. These include normal cognitive function, different states of wakefulness and sleep, in addition to pathological conditions, such as neurodegenerative diseases, the whole spectrum of neurocritical illnesses and encephalopathy. It is a fundamental principle in physiology and allied biomedical sciences that the results we obtain are only as robust as the methods we use. With this in mind, we also welcome the systematic assessments of the reliability of ^1^H MRS‐based creatine concentration measurements across different brain regions, reporting both within‐ (intra‐) and between‐ (inter‐)session reliability, in the study by Pratt et al. (2025). Their work identifies sources of error and presents new minimum detectable change data, contributing valuable methodological insights. This is an important contribution to the field, and we commend the authors on their work.

Inspired by the disciplines of psychometrics and clinimetrics, which focus on measurement methods in psychology and clinical medicine, respectively, we have introduced the term ‘physiolometrics’ to designate the field specifically concerned with the development and systematic assessment of measurement methods in physiology (Hartmann et al., 2023). Within this field, validity and reliability are the principal concepts used to evaluate the quality of a given method, which is ultimately crucial to the interpretation of any set of physiological data. Key domains pertain to the accuracy and precision of measurement methods, encompassed in the concepts of validity (i.e., the ability of a method accurately to reflect the true physiological variable) and reliability, referring to the consistency of repeated measurements (Hartmann et al., 2023; Schober et al., 2021). Other related domains include intra‐ and inter‐rater agreement assessments. However, physiolometrics remains a developing discipline, with no universal consensus on how best to define and apply its various terms, nor on which measures most appropriately reflect specific aspects of measurement quality. In this context, and with great respect for the important work by Pratt et al. (2025), we see their study as a vehicle for further discussion. The points we raise are by no means unique to their work, and indeed, when reflecting on our own previous publications, we recognize certain inconsistencies in our use of definitions and terms (Madsen et al., 2023; Mohammad & Hartmann, 2025; Mohammad, Hartmann, et al., 2025; Mohammad, Thomsen, et al., 2025; Olsen et al., 2022, 2021, 2024; Riberholt et al., 2021), underscoring the fact that physiolometrics is still a discipline in the making.

In their paper, Pratt et al. (2025) distinguish between reliability and repeatability, assessed both intra‐ and intersession, using Pearson's correlation coefficient, coefficient of variation (CV), intraclass correlation coefficient (ICC) and Bland–Altman plots. However, we consider reliability as an overarching concept, encompassing repeatability when referring to the consistency of measurements repeated in identical experimental conditions and reproducibility when referring to consistency across varying experimental conditions (Bartlett & Frost, 2008). Accordingly, appropriate terminology in this case could be either simply intra‐ and intersession reliability or, alternatively, repeatability and reproducibility for each of these assessments. Furthermore, the question remains of how each physiolometric measure should be interpreted. We posit that all the reported measures can be used to assess either repeatability or reproducibility, but they differ in whether they reflect these properties in absolute or relative terms.

Absolute reliability, whether referring to repeatability or to reproducibility, focuses on the magnitude or extent of measurement error, quantified in the same units as the measure of interest. This can be assessed by the standard error of measurement (SEM), which can be used to derive the so‐called smallest real difference (SRD), which represents the minimal change in a score that can be interpreted as a real difference, exceeding the expected measurement error (Vaz et al., 2013), and thus equivalent to the minimum detectable change reported here. Bland–Altman plots with derivation of bias and limits of agreement can also be used. In contrast, relative reliability provides information about the proportion of measurement error relative to the overall variation in the measure, typically expressed as a fraction or percentage. In the present study, this was assessed using the ICC and CV. The ICC is a unitless ratio of between‐subject variance to total variance, which appears to be particularly popular these days, perhaps owing to its convenient classification of reliability into categorical levels, such as poor, moderate, good and excellent (Koo & Li, 2016; Lee et al., 2012; Schober et al., 2021). However, the ICC has inherent limitations, including the sensitivity to variations both within and between groups, meaning that in a highly heterogeneous population, a high within‐group standard deviation could yield a high ICC regardless of real reliability of the method. In contrast, CV, which reflects the relationship between the within‐group standard deviation (i.e., the standard error of measurement) and the mean (Schober et al., 2021) is arguably a more useful and easily interpretable measure of relative reliability. In contrast, although widely used, Pearson's *r* quantifies correlation, but it should not be interpreted as a measure of reliability or agreement in the context of repeated measures. Primarily, one of the main assumptions is that each pair of *x–y* measurements must be independent (Schober & Schwarte, 2018). Thus, using Pearson's correlation coefficient for comparisons of the same variable would not provide information on reliability but would merely show the mathematical coupling between the measurements (Olsen et al., 2024). In our opinion, it should therefore be avoided entirely for this purpose. Furthermore, for all absolute and relative reliability estimates, we recommend reporting them alongside 95% confidence intervals to provide a more comprehensive assessment of measurement precision. To facilitate this, we have developed an R package that enables the calculation of 95% confidence intervals for the SRD, ICC and CV (Olsen et al., 2023).

Moreover, strict cut‐off values for acceptable and unacceptable or excellent and poor values are arbitrary and depend entirely on the research field, where a CV of 15% in one field could be acceptable and in others fatal (Hartmann et al., 2023). Therefore, reporting raw values (possibly together with contextually defined cut‐offs) might be more appropriate, enabling the reader to interpret the results independently and to make comparisons with other studies. Thus, in general, reliability studies should be careful only to use terms such as ‘excellent’, ‘moderate’, ‘poor’, ‘acceptable’ or ‘unacceptable’ in the assessment of reliability, which, in turn, could result in blindly accepting a threshold, as is often seen with an α for statistical hypothesis testing of 0.05 (Berg et al., 2024; Williams et al., 2023).

We welcome any future discussion in the pages of *Experimental Physiology* on how to define and apply different physiolometric terms and on which statistical tools are most appropriate for their assessment. However, despite the reservations we have raised regarding terminology, these do not pertain to the methods or results of the study by Pratt et al. (2025). As such, this stands out as an excellent study, and beyond semantics there is no doubt that the methods used and the results presented in their paper are both valid and reliable.

## AUTHOR CONTRIBUTIONS

Rie Skovly Thomsen: First draft; revisions. Milan Mohammad: Conception; revisions. Ronan M. G. Berg: First draft; revisions. Jacob Peter Hartmann: Supervision; revisions. Markus Harboe Olsen: Conception; first draft; revisions; supervision. All approved the final version of the manuscript and agree to be accountable for all aspects of the work in ensuring that questions related to the accuracy or integrity of any part of the work are appropriately investigated and resolved. All persons designated as authors qualify for authorship, and all those who qualify for authorship are listed.

## COMPETING INTEREST

None of the authors has any conflict of interest to declare.
